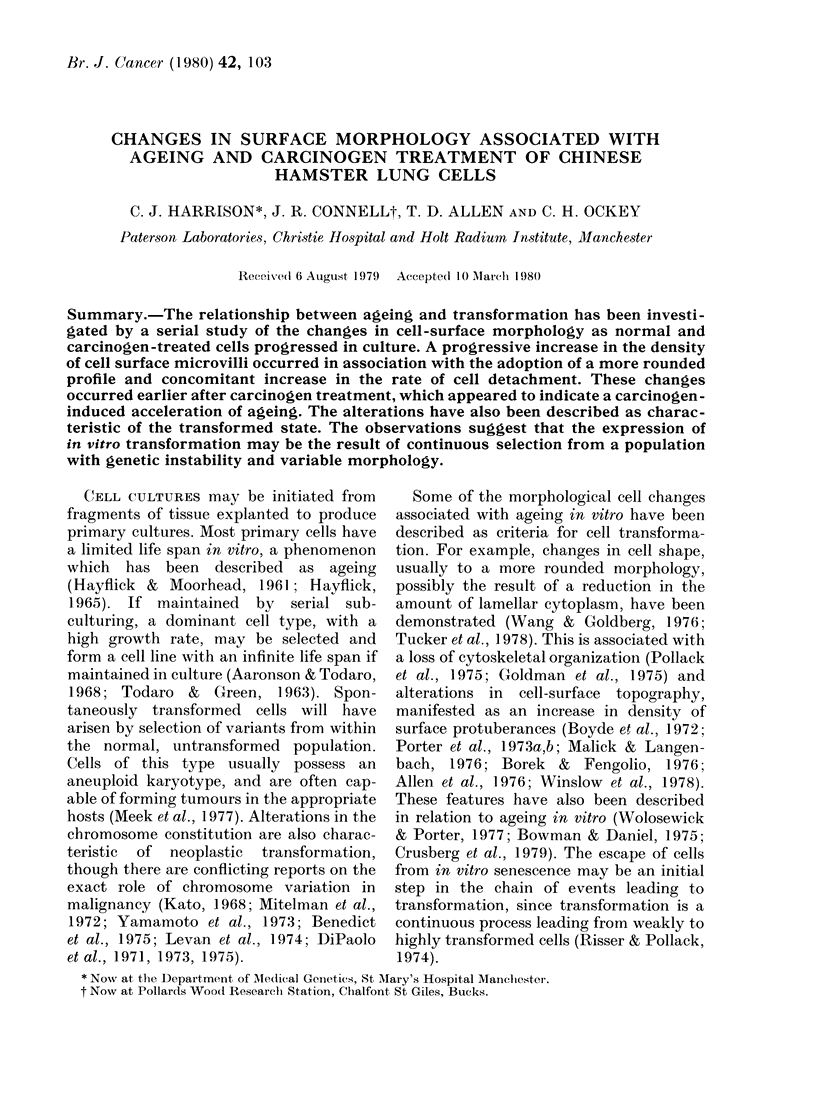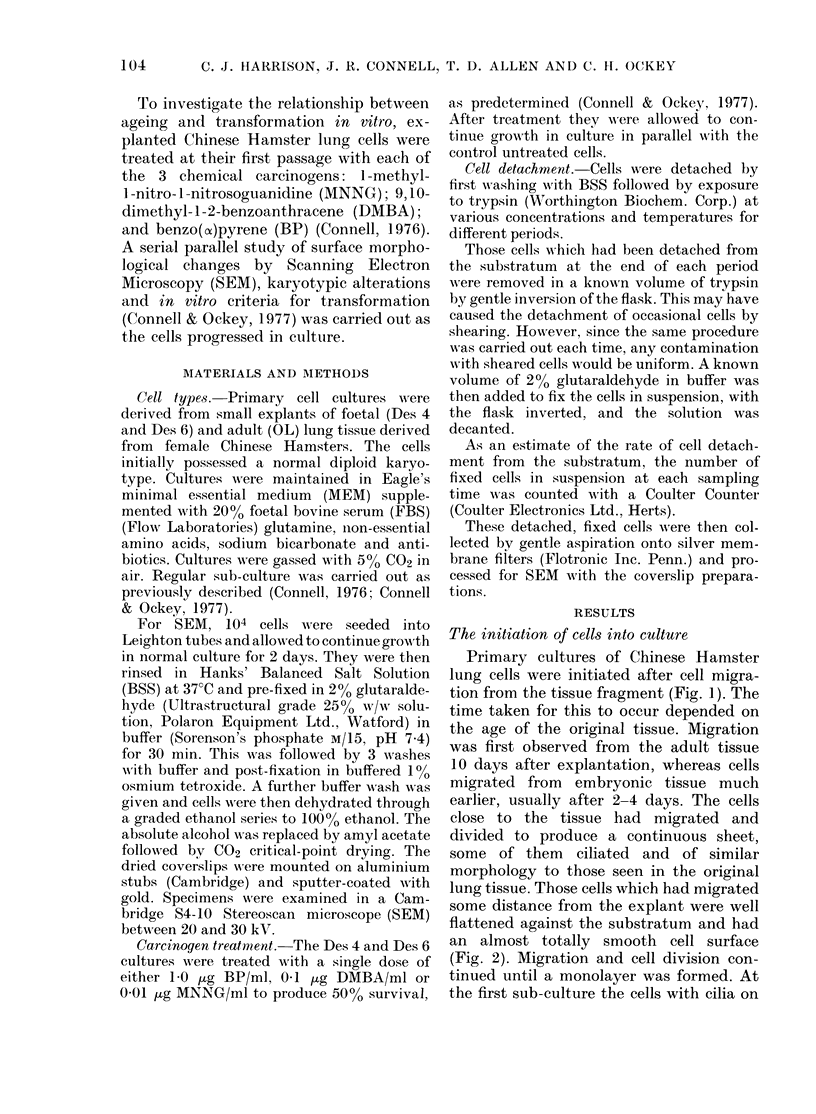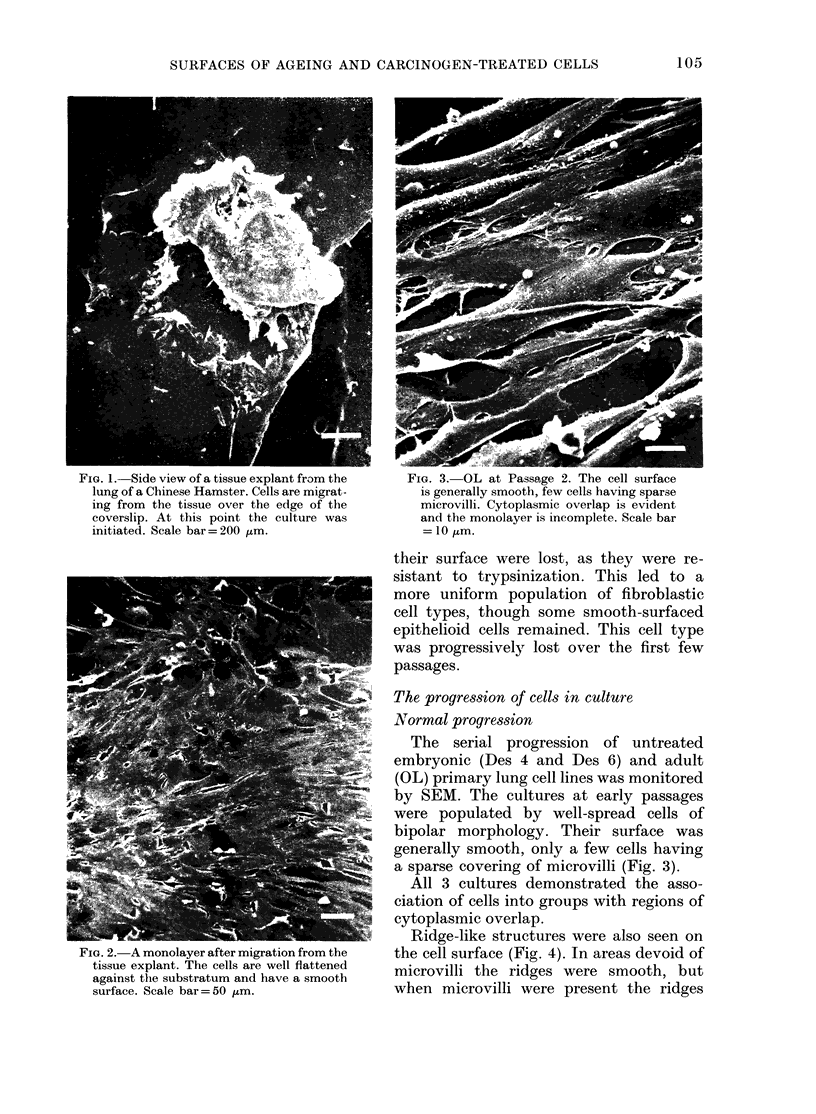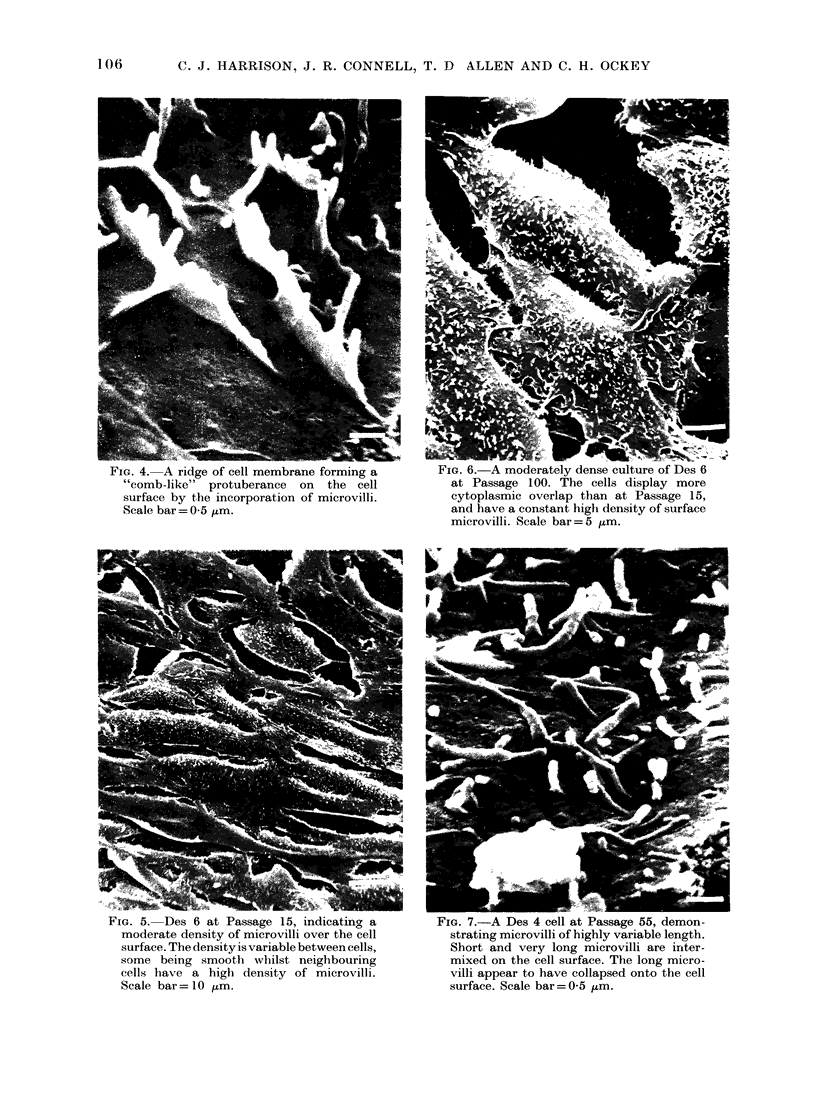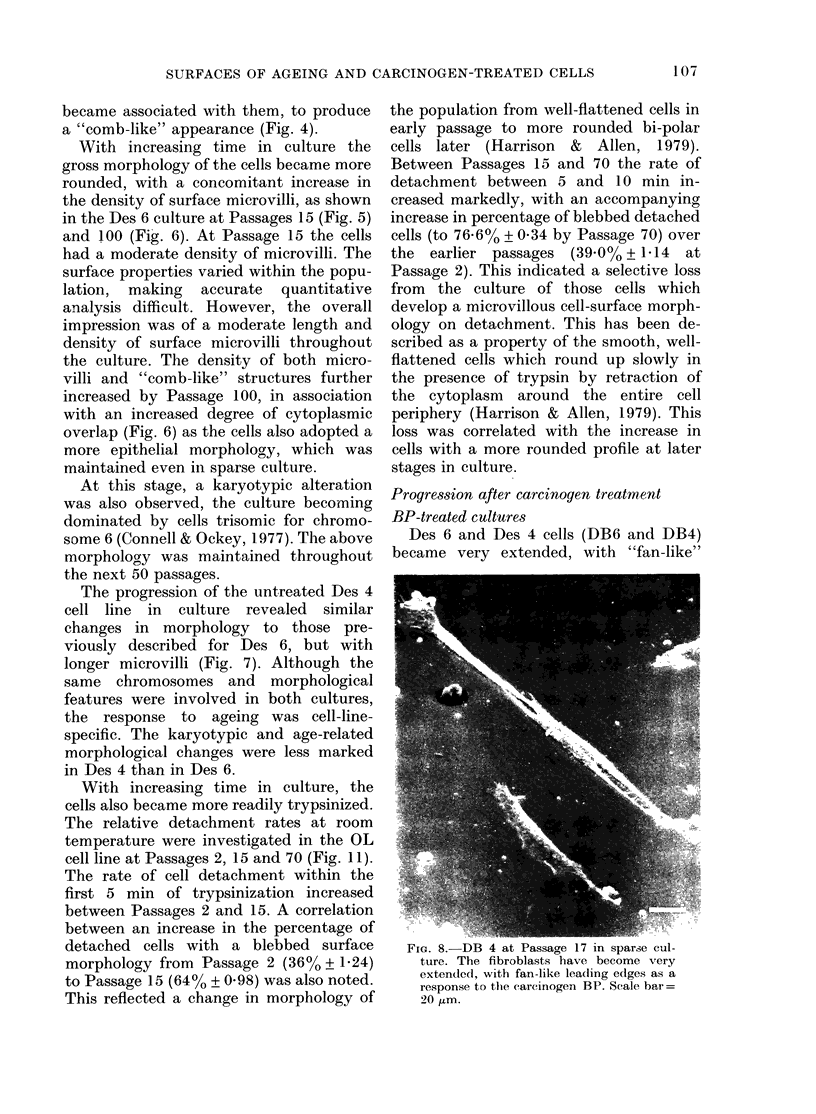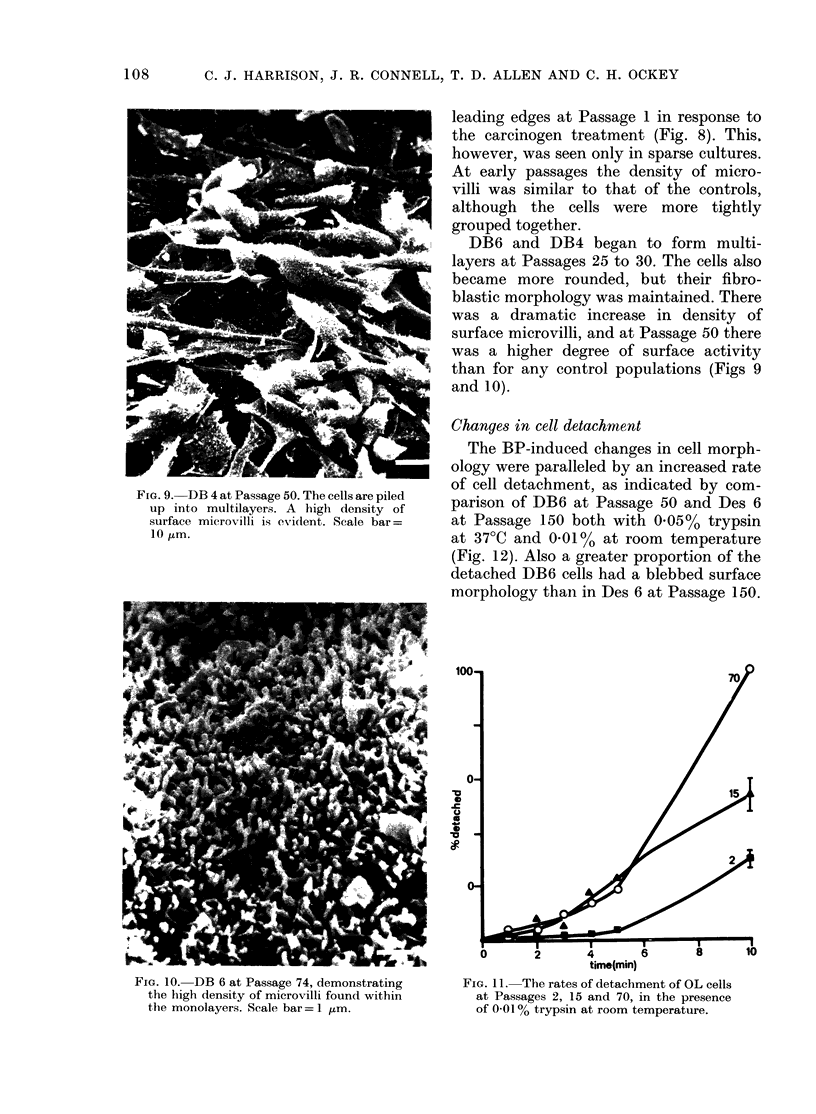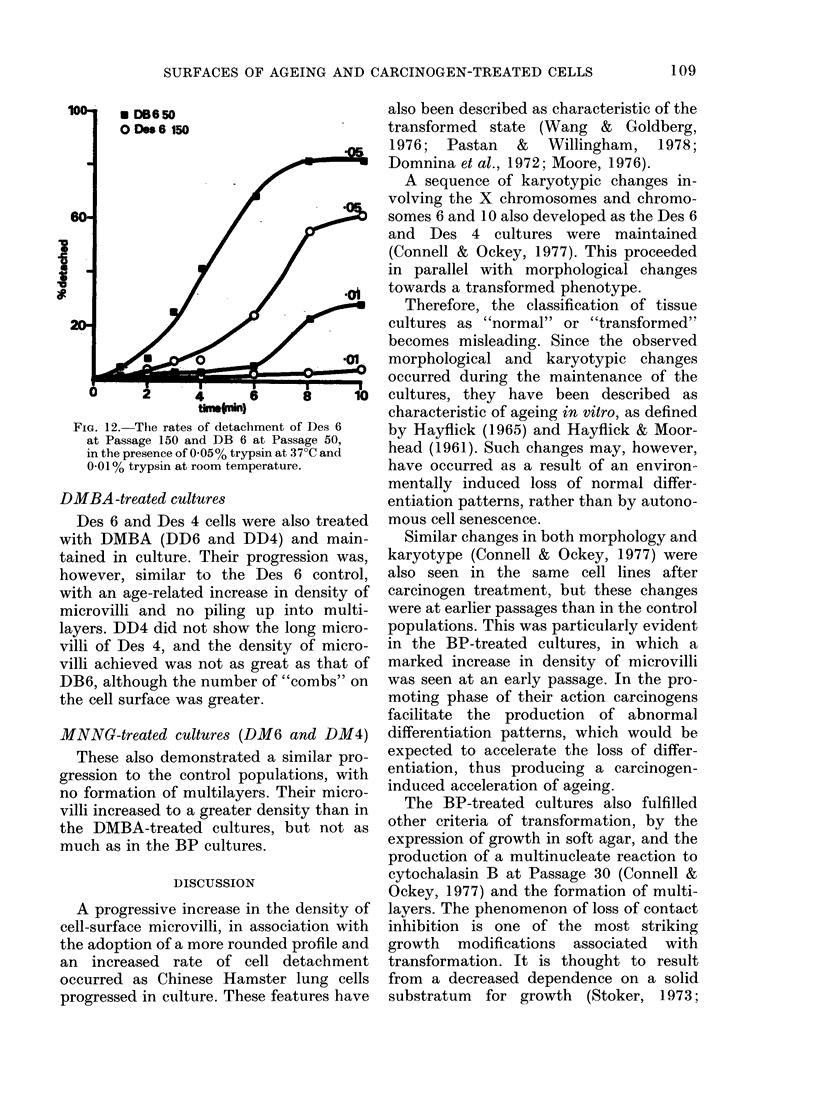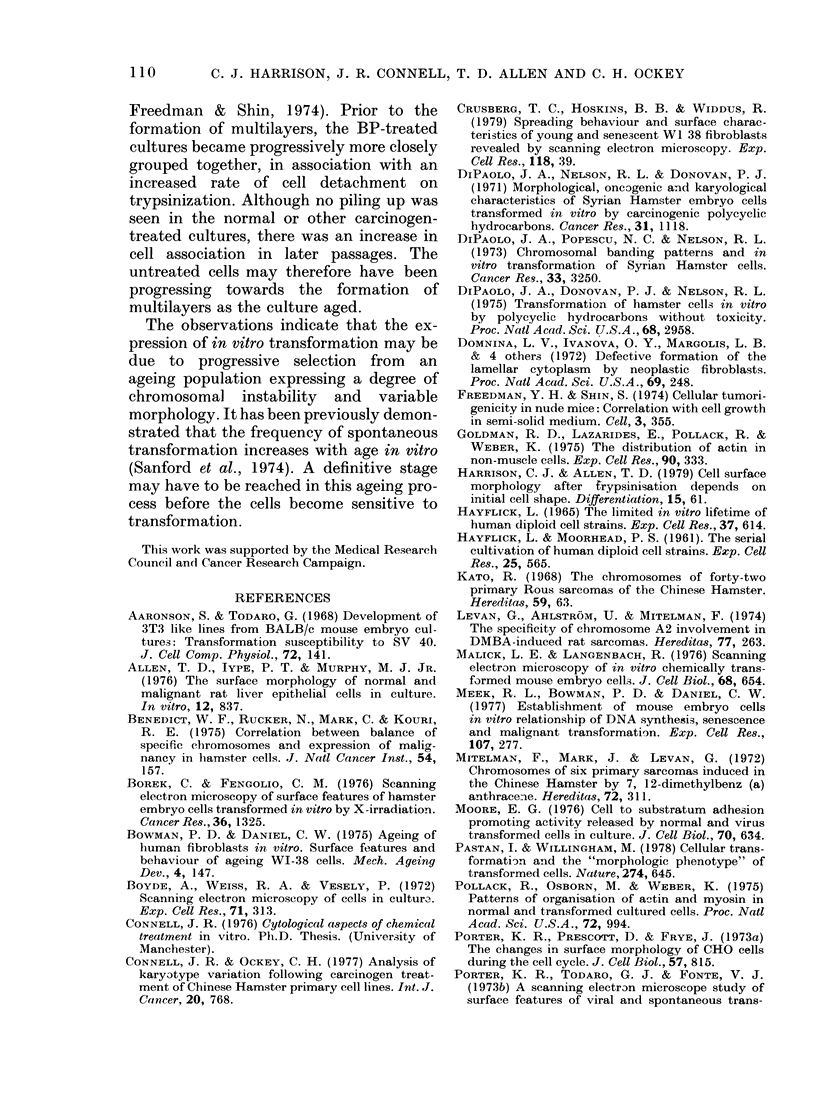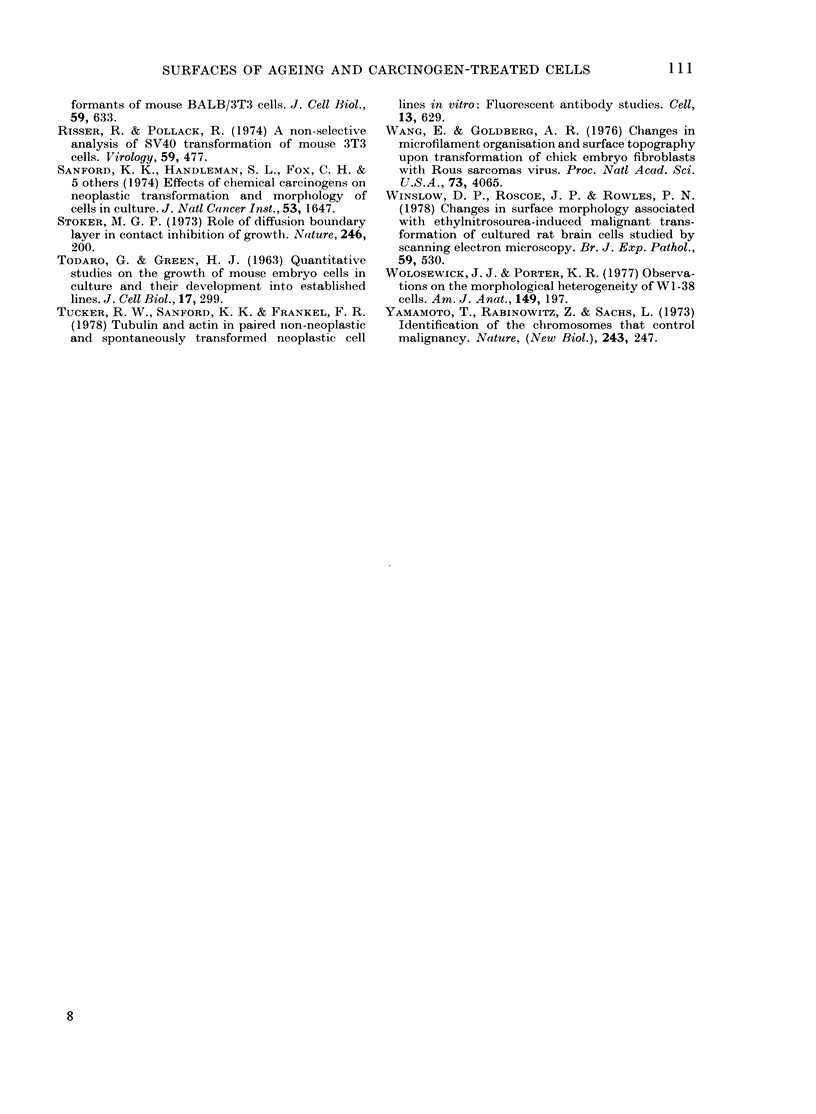# Changes in surface morphology associated with ageing and carcinogen treatment of Chinese hamster lung cells.

**DOI:** 10.1038/bjc.1980.208

**Published:** 1980-07

**Authors:** C. J. Harrison, J. R. Connell, T. D. Allen, C. H. Ockey

## Abstract

**Images:**


*Br. J. Cancer* (1980) **42**, 103

### CHANGES IN SURFACE MORPHOLOGY ASSOCIATED WITH AGEING AND CARCINOGEN TREATMENT OF CHINESE HAMSTER LUNG CELLS

C. J. HARRISON*, J. R. CONNELL†, T. D. ALLEN AND C. H. OCKEY

*Paterson Laboratories, Christie Hospital and Holt Radium Institute, Manchester*

Received 6 August 1979 Accepted 10 March 1980

**Summary.—The relationship between ageing and transformation has been investigated by a serial study of the changes in cell-surface morphology as normal and carcinogen-treated cells progressed in culture. A progressive increase in the density of cell surface microvilli occurred in association with the adoption of a more rounded profile and concomitant increase in the rate of cell detachment. These changes occurred earlier after carcinogen treatment, which appeared to indicate a carcinogen-induced acceleration of ageing. The alterations have also been described as characteristic of the transformed state. The observations suggest that the expression of *in vitro* transformation may be the result of continuous selection from a population with genetic instability and variable morphology.**

CELL CULTURES may be initiated from fragments of tissue explanted to produce primary cultures. Most primary cells have a limited life span *in vitro*, a phenomenon which has been described as ageing (Hayflick & Moorhead, 1961; Hayflick, 1965). If maintained by serial subculturing, a dominant cell type, with a high growth rate, may be selected and form a cell line with an infinite life span if maintained in culture (Aaronson & Todaro, 1968; Todaro & Green, 1963). Spontaneously transformed cells will have arisen by selection of variants from within the normal, untransformed population. Cells of this type usually possess an aneuploid karyotype, and are often capable of forming tumours in the appropriate hosts (Meek *et al.*, 1977). Alterations in the chromosome constitution are also characteristic of neoplastic transformation, though there are conflicting reports on the exact role of chromosome variation in malignancy (Kato, 1968; Mitelman *et al.*, 1972; Yamamoto *et al.*, 1973; Benedict *et al.*, 1975; Levan *et al.*, 1974; DiPaolo *et al.*, 1971, 1973, 1975).

Some of the morphological cell changes associated with ageing *in vitro* have been described as criteria for cell transformation. For example, changes in cell shape, usually to a more rounded morphology, possibly the result of a reduction in the amount of lamellar cytoplasm, have been demonstrated (Wang & Goldberg, 1976; Tucker *et al.*, 1978). This is associated with a loss of cytoskeletal organization (Pollack *et al.*, 1975; Goldman *et al.*, 1975) and alterations in cell-surface topography, manifested as an increase in density of surface protuberances (Boyde *et al.*, 1972; Porter *et al.*, 1973*a*,*b*; Malick & Langenbach, 1976; Borek & Fengolio, 1976; Allen *et al.*, 1976; Winslow *et al.*, 1978). These features have also been described in relation to ageing *in vitro* (Wolosewick & Porter, 1977; Bowman & Daniel, 1975; Crusberg *et al.*, 1979). The escape of cells from *in vitro* senescence may be an initial step in the chain of events leading to transformation, since transformation is a continuous process leading from weakly to highly transformed cells (Risser & Pollack, 1974).

* Now at the Department of Medical Genetics, St Mary's Hospital Manchester.
† Now at Pollards Wood Research Station, Chalfont St Giles, Bucks.

To investigate the relationship between ageing and transformation *in vitro*, explanted Chinese Hamster lung cells were treated at their first passage with each of the 3 chemical carcinogens: 1-methyl-1-nitro-1-nitrosoguanidine (MNNG); 9,10-dimethyl-1-2-benzoanthracene (DMBA); and benzo(α)pyrene (BP) (Connell, 1976). A serial parallel study of surface morphological changes by Scanning Electron Microscopy (SEM), karyotypic alterations and *in vitro* criteria for transformation (Connell & Ockey, 1977) was carried out as the cells progressed in culture.

### MATERIALS AND METHODS

*Cell types*.—Primary cell cultures were derived from small explants of foetal (Des 4 and Des 6) and adult (OL) lung tissue derived from female Chinese Hamsters. The cells initially possessed a normal diploid karyotype. Cultures were maintained in Eagle's minimal essential medium (MEM) supplemented with 20% foetal bovine serum (FBS) (Flow Laboratories) glutamine, non-essential amino acids, sodium bicarbonate and antibiotics. Cultures were gassed with 5% $CO_2$ in air. Regular sub-culture was carried out as previously described (Connell, 1976; Connell & Ockey, 1977).

For SEM, $10^4$ cells were seeded into Leighton tubes and allowed to continue growth in normal culture for 2 days. They were then rinsed in Hanks' Balanced Salt Solution (BSS) at 37°C and pre-fixed in 2% glutaraldehyde (Ultrastructural grade 25% w/w solution, Polaron Equipment Ltd., Watford) in buffer (Sorenson's phosphate M/15, pH 7·4) for 30 min. This was followed by 3 washes with buffer and post-fixation in buffered 1% osmium tetroxide. A further buffer wash was given and cells were then dehydrated through a graded ethanol series to 100% ethanol. The absolute alcohol was replaced by amyl acetate followed by $CO_2$ critical-point drying. The dried coverslips were mounted on aluminium stubs (Cambridge) and sputter-coated with gold. Specimens were examined in a Cambridge S4-10 Stereoscan microscope (SEM) between 20 and 30 kV.

*Carcinogen treatment*.—The Des 4 and Des 6 cultures were treated with a single dose of either 1·0 μg BP/ml, 0·1 μg DMBA/ml or 0·01 μg MNNG/ml to produce 50% survival, as predetermined (Connell & Ockey, 1977). After treatment they were allowed to continue growth in culture in parallel with the control untreated cells.

*Cell detachment*.—Cells were detached by first washing with BSS followed by exposure to trypsin (Worthington Biochem. Corp.) at various concentrations and temperatures for different periods.

Those cells which had been detached from the substratum at the end of each period were removed in a known volume of trypsin by gentle inversion of the flask. This may have caused the detachment of occasional cells by shearing. However, since the same procedure was carried out each time, any contamination with sheared cells would be uniform. A known volume of 2% glutaraldehyde in buffer was then added to fix the cells in suspension, with the flask inverted, and the solution was decanted.

As an estimate of the rate of cell detachment from the substratum, the number of fixed cells in suspension at each sampling time was counted with a Coulter Counter (Coulter Electronics Ltd., Herts).

These detached, fixed cells were then collected by gentle aspiration onto silver membrane filters (Flotronic Inc. Penn.) and processed for SEM with the coverslip preparations.

### RESULTS

### *The initiation of cells into culture*

Primary cultures of Chinese Hamster lung cells were initiated after cell migration from the tissue fragment (Fig. 1). The time taken for this to occur depended on the age of the original tissue. Migration was first observed from the adult tissue 10 days after explantation, whereas cells migrated from embryonic tissue much earlier, usually after 2–4 days. The cells close to the tissue had migrated and divided to produce a continuous sheet, some of them ciliated and of similar morphology to those seen in the original lung tissue. Those cells which had migrated some distance from the explant were well flattened against the substratum and had an almost totally smooth cell surface (Fig. 2). Migration and cell division continued until a monolayer was formed. At the first sub-culture the cells with cilia on

FIG. 1.—Side view of a tissue explant from the lung of a Chinese Hamster. Cells are migrating from the tissue over the edge of the coverslip. At this point the culture was initiated. Scale bar = 200 $\mu$m.

FIG. 2.—A monolayer after migration from the tissue explant. The cells are well flattened against the substratum and have a smooth surface. Scale bar = 50 $\mu$m.

FIG. 3.—OL at Passage 2. The cell surface is generally smooth, few cells having sparse microvilli. Cytoplasmic overlap is evident and the monolayer is incomplete. Scale bar = 10 $\mu$m.

their surface were lost, as they were resistant to trypsinization. This led to a more uniform population of fibroblastic cell types, though some smooth-surfaced epithelioid cells remained. This cell type was progressively lost over the first few passages.

*The progression of cells in culture*

*Normal progression*

The serial progression of untreated embryonic (Des 4 and Des 6) and adult (OL) primary lung cell lines was monitored by SEM. The cultures at early passages were populated by well-spread cells of bipolar morphology. Their surface was generally smooth, only a few cells having a sparse covering of microvilli (Fig. 3).

All 3 cultures demonstrated the association of cells into groups with regions of cytoplasmic overlap.

Ridge-like structures were also seen on the cell surface (Fig. 4). In areas devoid of microvilli the ridges were smooth, but when microvilli were present the ridges

Fig. 4.—A ridge of cell membrane forming a "comb-like" protuberance on the cell surface by the incorporation of microvilli. Scale bar = 0·5 μm.

Fig. 6.—A moderately dense culture of Des 6 at Passage 100. The cells display more cytoplasmic overlap than at Passage 15, and have a constant high density of surface microvilli. Scale bar = 5 μm.

Fig. 5.—Des 6 at Passage 15, indicating a moderate density of microvilli over the cell surface. The density is variable between cells, some being smooth whilst neighbouring cells have a high density of microvilli. Scale bar = 10 μm.

Fig. 7.—A Des 4 cell at Passage 55, demonstrating microvilli of highly variable length. Short and very long microvilli are intermixed on the cell surface. The long microvilli appear to have collapsed onto the cell surface. Scale bar = 0·5 μm.

became associated with them, to produce a "comb-like" appearance (Fig. 4).

With increasing time in culture the gross morphology of the cells became more rounded, with a concomitant increase in the density of surface microvilli, as shown in the Des 6 culture at Passages 15 (Fig. 5) and 100 (Fig. 6). At Passage 15 the cells had a moderate density of microvilli. The surface properties varied within the population, making accurate quantitative analysis difficult. However, the overall impression was of a moderate length and density of surface microvilli throughout the culture. The density of both microvilli and "comb-like" structures further increased by Passage 100, in association with an increased degree of cytoplasmic overlap (Fig. 6) as the cells also adopted a more epithelial morphology, which was maintained even in sparse culture.

At this stage, a karyotypic alteration was also observed, the culture becoming dominated by cells trisomic for chromosome 6 (Connell & Ockey, 1977). The above morphology was maintained throughout the next 50 passages.

The progression of the untreated Des 4 cell line in culture revealed similar changes in morphology to those previously described for Des 6, but with longer microvilli (Fig. 7). Although the same chromosomes and morphological features were involved in both cultures, the response to ageing was cell-line-specific. The karyotypic and age-related morphological changes were less marked in Des 4 than in Des 6.

With increasing time in culture, the cells also became more readily trypsinized. The relative detachment rates at room temperature were investigated in the OL cell line at Passages 2, 15 and 70 (Fig. 11). The rate of cell detachment within the first 5 min of trypsinization increased between Passages 2 and 15. A correlation between an increase in the percentage of detached cells with a blebbed surface morphology from Passage 2 ($36\% \pm 1{\cdot}24$) to Passage 15 ($64\% \pm 0{\cdot}98$) was also noted. This reflected a change in morphology of the population from well-flattened cells in early passage to more rounded bi-polar cells later (Harrison & Allen, 1979). Between Passages 15 and 70 the rate of detachment between 5 and 10 min increased markedly, with an accompanying increase in percentage of blebbed detached cells (to $76{\cdot}6\% \pm 0{\cdot}34$ by Passage 70) over the earlier passages ($39{\cdot}0\% \pm 1{\cdot}14$ at Passage 2). This indicated a selective loss from the culture of those cells which develop a microvillous cell-surface morphology on detachment. This has been described as a property of the smooth, well-flattened cells which round up slowly in the presence of trypsin by retraction of the cytoplasm around the entire cell periphery (Harrison & Allen, 1979). This loss was correlated with the increase in cells with a more rounded profile at later stages in culture.

*Progression after carcinogen treatment*

*BP-treated cultures*

Des 6 and Des 4 cells (DB6 and DB4) became very extended, with "fan-like"

Fig. 8.—DB 4 at Passage 17 in sparse culture. The fibroblasts have become very extended, with fan-like leading edges as a response to the carcinogen BP. Scale bar = 20 μm.

leading edges at Passage 1 in response to the carcinogen treatment (Fig. 8). This, however, was seen only in sparse cultures. At early passages the density of microvilli was similar to that of the controls, although the cells were more tightly grouped together.

DB6 and DB4 began to form multilayers at Passages 25 to 30. The cells also became more rounded, but their fibroblastic morphology was maintained. There was a dramatic increase in density of surface microvilli, and at Passage 50 there was a higher degree of surface activity than for any control populations (Figs 9 and 10).

FIG. 9.—DB 4 at Passage 50. The cells are piled up into multilayers. A high density of surface microvilli is evident. Scale bar = 10 μm.

*Changes in cell detachment*

The BP-induced changes in cell morphology were paralleled by an increased rate of cell detachment, as indicated by comparison of DB6 at Passage 50 and Des 6 at Passage 150 both with 0·05% trypsin at 37°C and 0·01% at room temperature (Fig. 12). Also a greater proportion of the detached DB6 cells had a blebbed surface morphology than in Des 6 at Passage 150.

FIG. 10.—DB 6 at Passage 74, demonstrating the high density of microvilli found within the monolayers. Scale bar = 1 μm.

FIG. 11.—The rates of detachment of OL cells at Passages 2, 15 and 70, in the presence of 0·01% trypsin at room temperature.

FIG. 12.—The rates of detachment of Des 6 at Passage 150 and DB 6 at Passage 50, in the presence of 0·05% trypsin at 37°C and 0·01% trypsin at room temperature.

*DMBA-treated cultures*

Des 6 and Des 4 cells were also treated with DMBA (DD6 and DD4) and maintained in culture. Their progression was, however, similar to the Des 6 control, with an age-related increase in density of microvilli and no piling up into multilayers. DD4 did not show the long microvilli of Des 4, and the density of microvilli achieved was not as great as that of DB6, although the number of "combs" on the cell surface was greater.

*MNNG-treated cultures (DM6 and DM4)*

These also demonstrated a similar progression to the control populations, with no formation of multilayers. Their microvilli increased to a greater density than in the DMBA-treated cultures, but not as much as in the BP cultures.

### DISCUSSION

A progressive increase in the density of cell-surface microvilli, in association with the adoption of a more rounded profile and an increased rate of cell detachment occurred as Chinese Hamster lung cells progressed in culture. These features have also been described as characteristic of the transformed state (Wang & Goldberg, 1976; Pastan & Willingham, 1978; Domnina *et al.*, 1972; Moore, 1976).

A sequence of karyotypic changes involving the X chromosomes and chromosomes 6 and 10 also developed as the Des 6 and Des 4 cultures were maintained (Connell & Ockey, 1977). This proceeded in parallel with morphological changes towards a transformed phenotype.

Therefore, the classification of tissue cultures as "normal" or "transformed" becomes misleading. Since the observed morphological and karyotypic changes occurred during the maintenance of the cultures, they have been described as characteristic of ageing *in vitro*, as defined by Hayflick (1965) and Hayflick & Moorhead (1961). Such changes may, however, have occurred as a result of an environmentally induced loss of normal differentiation patterns, rather than by autonomous cell senescence.

Similar changes in both morphology and karyotype (Connell & Ockey, 1977) were also seen in the same cell lines after carcinogen treatment, but these changes were at earlier passages than in the control populations. This was particularly evident in the BP-treated cultures, in which a marked increase in density of microvilli was seen at an early passage. In the promoting phase of their action carcinogens facilitate the production of abnormal differentiation patterns, which would be expected to accelerate the loss of differentiation, thus producing a carcinogen-induced acceleration of ageing.

The BP-treated cultures also fulfilled other criteria of transformation, by the expression of growth in soft agar, and the production of a multinucleate reaction to cytochalasin B at Passage 30 (Connell & Ockey, 1977) and the formation of multilayers. The phenomenon of loss of contact inhibition is one of the most striking growth modifications associated with transformation. It is thought to result from a decreased dependence on a solid substratum for growth (Stoker, 1973;

Freedman & Shin, 1974). Prior to the formation of multilayers, the BP-treated cultures became progressively more closely grouped together, in association with an increased rate of cell detachment on trypsinization. Although no piling up was seen in the normal or other carcinogen-treated cultures, there was an increase in cell association in later passages. The untreated cells may therefore have been progressing towards the formation of multilayers as the culture aged.

The observations indicate that the expression of *in vitro* transformation may be due to progressive selection from an ageing population expressing a degree of chromosomal instability and variable morphology. It has been previously demonstrated that the frequency of spontaneous transformation increases with age *in vitro* (Sanford *et al.*, 1974). A definitive stage may have to be reached in this ageing process before the cells become sensitive to transformation.

This work was supported by the Medical Research Council and Cancer Research Campaign.

### REFERENCES

AARONSON, S. & TODARO, G. (1968) Development of 3T3 like lines from BALB/c mouse embryo cultures: Transformation susceptibility to SV 40. *J. Cell Comp. Physiol.*, **72**, 141.

ALLEN, T. D., IYPE, P. T. & MURPHY, M. J. JR. (1976) The surface morphology of normal and malignant rat liver epithelial cells in culture. *In vitro*, **12**, 837.

BENEDICT, W. F., RUCKER, N., MARK, C. & KOURI, R. E. (1975) Correlation between balance of specific chromosomes and expression of malignancy in hamster cells. *J. Natl Cancer Inst.*, **54**, 157.

BOREK, C. & FENGOLIO, C. M. (1976) Scanning electron microscopy of surface features of hamster embryo cells transformed *in vitro* by X-irradiation. *Cancer Res.*, **36**, 1325.

BOWMAN, P. D. & DANIEL, C. W. (1975) Ageing of human fibroblasts *in vitro*. Surface features and behaviour of ageing WI-38 cells. *Mech. Ageing Dev.*, **4**, 147.

BOYDE, A., WEISS, R. A. & VESELY, P. (1972) Scanning electron microscopy of cells in culture. *Exp. Cell Res.*, **71**, 313.

CONNELL, J. R. (1976) *Cytological aspects of chemical treatment* in vitro. Ph.D. Thesis. (University of Manchester).

CONNELL, J. R. & OCKEY, C. H. (1977) Analysis of karyotype variation following carcinogen treatment of Chinese Hamster primary cell lines. *Int. J. Cancer*, **20**, 768.

CRUSBERG, T. C., HOSKINS, B. B. & WIDDUS, R. (1979) Spreading behaviour and surface characteristics of young and senescent W1 38 fibroblasts revealed by scanning electron microscopy. *Exp. Cell Res.*, **118**, 39.

DIPAOLO, J. A., NELSON, R. L. & DONOVAN, P. J. (1971) Morphological, oncogenic and karyological characteristics of Syrian Hamster embryo cells transformed *in vitro* by carcinogenic polycyclic hydrocarbons. *Cancer Res.*, **31**, 1118.

DIPAOLO, J. A., POPESCU, N. C. & NELSON, R. L. (1973) Chromosomal banding patterns and *in vitro* transformation of Syrian Hamster cells. *Cancer Res.*, **33**, 3250.

DIPAOLO, J. A., DONOVAN, P. J. & NELSON, R. L. (1975) Transformation of hamster cells *in vitro* by polycyclic hydrocarbons without toxicity. *Proc. Natl Acad. Sci. U.S.A.*, **68**, 2958.

DOMNINA, L. V., IVANOVA, O. Y., MARGOLIS, L. B. & 4 others (1972) Defective formation of the lamellar cytoplasm by neoplastic fibroblasts. *Proc. Natl Acad. Sci. U.S.A.*, **69**, 248.

FREEDMAN, Y. H. & SHIN, S. (1974) Cellular tumorigenicity in nude mice: Correlation with cell growth in semi-solid medium. *Cell*, **3**, 355.

GOLDMAN, R. D., LAZARIDES, E., POLLACK, R. & WEBER, K. (1975) The distribution of actin in non-muscle cells. *Exp. Cell Res.*, **90**, 333.

HARRISON, C. J. & ALLEN, T. D. (1979) Cell surface morphology after trypsinisation depends on initial cell shape. *Differentiation*, **15**, 61.

HAYFLICK, L. (1965) The limited *in vitro* lifetime of human diploid cell strains. *Exp. Cell Res.*, **37**, 614.

HAYFLICK, L. & MOORHEAD, P. S. (1961). The serial cultivation of human diploid cell strains. *Exp. Cell Res.*, **25**, 565.

KATO, R. (1968) The chromosomes of forty-two primary Rous sarcomas of the Chinese Hamster. *Hereditas*, **59**, 63.

LEVAN, G., AHLSTRÖM, U. & MITELMAN, F. (1974) The specificity of chromosome A2 involvement in DMBA-induced rat sarcomas. *Hereditas*, **77**, 263.

MALICK, L. E. & LANGENBACH, R. (1976) Scanning electron microscopy of *in vitro* chemically transformed mouse embryo cells. *J. Cell Biol.*, **68**, 654.

MEEK, R. L., BOWMAN, P. D. & DANIEL, C. W. (1977) Establishment of mouse embryo cells *in vitro* relationship of DNA synthesis, senescence and malignant transformation. *Exp. Cell Res.*, **107**, 277.

MITELMAN, F., MARK, J. & LEVAN, G. (1972) Chromosomes of six primary sarcomas induced in the Chinese Hamster by 7, 12-dimethylbenz (a) anthracene. *Hereditas*, **72**, 311.

MOORE, E. G. (1976) Cell to substratum adhesion promoting activity released by normal and virus transformed cells in culture. *J. Cell Biol.*, **70**, 634.

PASTAN, I. & WILLINGHAM, M. (1978) Cellular transformation and the "morphologic phenotype" of transformed cells. *Nature*, **274**, 645.

POLLACK, R., OSBORN, M. & WEBER, K. (1975) Patterns of organisation of actin and myosin in normal and transformed cultured cells. *Proc. Natl Acad. Sci. U.S.A.*, **72**, 994.

PORTER, K. R., PRESCOTT, D. & FRYE, J. (1973*a*) The changes in surface morphology of CHO cells during the cell cycle. *J. Cell Biol.*, **57**, 815.

PORTER, K. R., TODARO, G. J. & FONTE, V. J. (1973*b*) A scanning electron microscope study of surface features of viral and spontaneous transformants of mouse BALB/3T3 cells. *J. Cell Biol.*, **59**, 633.

RISSER, R. & POLLACK, R. (1974) A non-selective analysis of SV40 transformation of mouse 3T3 cells. *Virology*, **59**, 477.

SANFORD, K. K., HANDLEMAN, S. L., FOX, C. H. & 5 others (1974) Effects of chemical carcinogens on neoplastic transformation and morphology of cells in culture. *J. Natl Cancer Inst.*, **53**, 1647.

STOKER, M. G. P. (1973) Role of diffusion boundary layer in contact inhibition of growth. *Nature*, **246**, 200.

TODARO, G. & GREEN, H. J. (1963) Quantitative studies on the growth of mouse embryo cells in culture and their development into established lines. *J. Cell Biol.*, **17**, 299.

TUCKER, R. W., SANFORD, K. K. & FRANKEL, F. R. (1978) Tubulin and actin in paired non-neoplastic and spontaneously transformed neoplastic cell lines *in vitro*: Fluorescent antibody studies. *Cell*, **13**, 629.

WANG, E. & GOLDBERG, A. R. (1976) Changes in microfilament organisation and surface topography upon transformation of chick embryo fibroblasts with Rous sarcomas virus. *Proc. Natl Acad. Sci. U.S.A.*, **73**, 4065.

WINSLOW, D. P., ROSCOE, J. P. & ROWLES, P. N. (1978) Changes in surface morphology associated with ethylnitrosourea-induced malignant transformation of cultured rat brain cells studied by scanning electron microscopy. *Br. J. Exp. Pathol.*, **59**, 530.

WOLOSEWICK, J. J. & PORTER, K. R. (1977) Observations on the morphological heterogeneity of W1-38 cells. *Am. J. Anat.*, **149**, 197.

YAMAMOTO, T., RABINOWITZ, Z. & SACHS, L. (1973) Identification of the chromosomes that control malignancy. *Nature*, (*New Biol.*), **243**, 247.